# Alterations in White Matter Microstructure as Vulnerability Factors and Acquired Signs of Traffic Accident-Induced PTSD

**DOI:** 10.1371/journal.pone.0083473

**Published:** 2013-12-13

**Authors:** Yawen Sun, Zhen Wang, Weina Ding, Jieqing Wan, Zhiguo Zhuang, Yong Zhang, Yijun Liu, Yan Zhou, Jianrong Xu

**Affiliations:** 1 Department of Radiology, Ren Ji Hospital, School of Medicine, Shanghai Jiao Tong University, Shanghai, P.R. China; 2 Shanghai Mental Health Center, School of Medicine, Shanghai Jiao Tong University, Shanghai, P.R. China; 3 Department of Neurosurgery, Ren Ji Hospital, School of Medicine, Shanghai Jiao Tong University, Shanghai, P.R. China; 4 Applied Science Laboratory, GE Healthcare, Shanghai, P.R. China; 5 Department of Psychiatry, University of Florida College of Medicine, Gainesville, Florida, United States of America; University of Electronic Science and Technology of China, China

## Abstract

It remains unclear whether white matter (WM) changes found in post-traumatic stress disorder (PTSD) patients are stress-induced or precursors for vulnerability. The current study aimed to identify susceptibility factors relating to the development of PTSD and to examine the ability of these factors to predict the course of longitudinal PTSD. Sixty two victims who had experienced traffic accidents underwent diffusion tensor imaging using a 3.0T MRI system within 2 days after their accidents. Of these, 21 were diagnosed with PTSD at 1 or 6 months using the Clinician-Administered Ptsd Scale (CAPS). Then, 11 trauma-exposed victims with PTSD underwent the second MRI scan. Compared with the victims without PTSD, the victims with PTSD showed decreased fractional anisotropy (FA) in WM of the anterior cingulate cortex, ventromedial prefrontal cortex (vmPFC), temporal lobes and midbrain, and increased mean diffusivity (MD) in the vmPFC within 2 days after the traumatic event. Importantly, decreased FA of the vmPFC in the acute phase predicted greater future CAPS scores. In addition, we found decreased FA in the insula in the follow-up scan in the victims with PTSD, which correlated with the decreased FA of the vmPFC in their baseline scan. These results suggested that the WM might have changed within 2 days after the traumatic event in the individuals who would later develop PTSD. Furthermore, decreased FA of the vmPFC could be a possible vulnerability marker predicting future development of PTSD and may provide an outcome prediction of the acquired signs.

## Introduction

Post-traumatic stress disorder (PTSD) is a highly prevalent problem among military personnel and veterans [1]. In the general population, traffic accidents are a more common form of trauma event which greatly increase the risk of developing PTSD. Data from WHO demonstrated that 20 to 50 million people are injured or disabled in road collisions over the course of one year [2]. These accidents are frequently followed by PTSD at about six months in 8.5–23.1% of victims [3,4]. However, many victims do not develop the symptoms that qualify them for the diagnosis of PTSD. The causal relationship between the traumatic event and the subsequent onset of PTSD remains unclear.

A number of neuroimaging studies have reported both structural and functional brain abnormalities associated with PTSD, particularly in the hippocampus, amygdala, anterior cingulate cortex (ACC), insula, and medial prefrontal cortex (mPFC) [5,6,7]. Most of the previous studies considered the structural and functional changes to be a consequence of suffering from PTSD due to excessive stress [8,9]. More recently, researchers have begun to focus on identifying the neural markers which can suggest susceptibility to PTSD. For instance, smaller hippocampal volume and hyper-responsiveness in the dorsal ACC were found to be a familial risk factor for the development of PTSD in a study of monozygotic twins [10,11]. However, a longitudinal structural MRI (sMRI) study in trauma survivors with PTSD failed to find smaller hippocampal volume as a necessary risk factor for developing PTSD [12]. In another longitudinal study of sMRI obtained before and after the earthquake, smaller gray matter volume (GMV) in the ACC was found to be related to the vulnerability for developing PTSD symptoms [13], while decreased GMV in the orbitofrontal cortex (OFC) represented an acquired sign of PTSD symptoms. However, all subjects in the longitudinal study were diagnosed as normal, and the relationship between vulnerability factors and the acquired signs of the PTSD was not examined [13]. A prospective study showed that low-frequency connectivity of the posterior cingulate/precuneus (PCC) with the amygdala at 5–6 weeks post-trauma could predict future PTSD symptoms [14]. However, the subsequent functional brain changes remained unclear. 

The present study therefore attempted to identify the possible susceptibility factors relating to the development of PTSD and examine the ability of these factors to predict the course of longitudinal PTSD. In fact, previous studies from our research group showed that, during the resting-state, early-detected changes (within 2 days after a traffic accident) in functional connectivity (FC) of the PCC with the mPFC, hippocampus and amygdala might be used as neural markers showing a predisposition for development of PTSD in these patients [15,16]. To the best of our knowledge, no study using diffusion tensor imaging (DTI) has investigated whether the white matter (WM) changes are the consequence of the traumatic stress or a predisposing risk factor for developing PTSD soon after traffic accident-induced trauma. In this study, voxel-based analysis (VBA) was employed to analyze the DTI data, which has the advantage of detecting WM changes through the entire brain on a voxel by voxel basis. We examined WM changes within 2 days after the accident in victims who had experienced acute traumatic events. The clinician-administered PTSD Scale (CAPS) conferred PTSD diagnoses at 1 month and 6 months later [17]. The trauma-exposed victims with PTSD underwent a second DTI scan at the time of diagnosis. Because our previous studies identified early altered resting-state functional connectivity in the mPFC, hippocampus and amygdala as a possible vulnerability factor [15,16], we hypothesized that altered FA or MD would be found in these WM region as precursors for vulnerability. We predicted that the follow-up assessment would detect longitudinal altered WM changes that represented an acquired sign in trauma-exposed victims with PTSD, because some longitudinal studies demonstrated significant difference in brain structural changes between baseline and follow-up by stress induced [13,18]. An additional aim was to identify the baseline predictors which would be associated with greater future CAPS changes [14,16]. 

## Materials and Methods

### Participants

Participants were recruited from patients admitted to the Emergency Department of Ren Ji Hospital following a traffic accident. During the first assessment session, participants without significant head injuries or loss of consciousness longer than several seconds completed a psychiatric clinical interview using the Mini-International Neuropsychiatric Interview (M.I.N.I) [19], and the acute stress disorder inventory (ASDI) [20] methods. M.I.N.I. served to evaluate current and past psychiatric Axis I comorbidity in all participants [19]. ASDI served to evaluate acute stress disorder symptoms [20]. In order to improve the positive predictive power of the participants developing PTSD in the future, we recruited subjects with ASDI scores greater than 3 [21]. A total of sixty-two right-handed (Male = 32; Female = 30) victims of car accidents were included in the subsequent study. After the interview, each participant had an MRI scan of the whole brain. All of the baseline evaluation and MRI scans were completed within 2 days of the accident. The next evaluations took place at 1 and 6 months after the accident, and were completed by 50 participants at 1 month and 46 participants at 6 months. The evaluation included the previous psychometric measures for PTSD diagnosis and symptom severity using the CAPS [17]. Of the 62 car-accident victims, 21 trauma-exposed victims met the criteria for diagnosis of PTSD as assessed using CAPS at 1 or 6 months following the accident; 17 age- and gender-matched trauma-exposed victims who did not develop PTSD at 6 months after trauma were selected as the control group. Eleven trauma-exposed victims with PTSD had the second MRI evaluation when they were diagnosed at 1 or 6 months later, but only 5 trauma-exposed victims without PTSD completed the second MRI scan at 6 months after the accident. 

We also recruited 22 right-handed healthy age- and gender-matched control subjects unaffected by trauma from the community. All 22 were interviewed by trained psychologists using the M.I.N.I. and underwent a whole brain MRI scan. The healthy controls and trauma-exposed victims without PTSD were compared in order to explore altered WM regions which correlated with the effect of the traumatic events. A comparison between the healthy controls and trauma-exposed victims with PTSD were also conducted.

The exclusion criteria were: (1) ages < 18 or > 60 years old, education < 9 years; (2) ASDI < 3; (3) significant head injuries (i.e., abnormalities on conventional MRI, neurological abnormality during emergency department evaluation, or loss of consciousness longer than several seconds during the accident based on self-report by the participant); (4) a history of neurological disorders; (5) current and past psychiatric Axis I disorders, as assessed using the M.I.N.I [19]; (6) substance abuse (drug or alcohol abuse/dependence within 6 months prior to accident); (7) medications (using psychotropics within 4 weeks prior to MRI scan); (8) MRI scan contraindications (e.g., floating metallic bodies, severe claustrophobia).

The current study was approved by the Research Ethics Committee of the Ren Ji Hospital, School of Medicine, Shanghai Jiao Tong University, and written informed consent was obtained from each subject before participation. All procedures were in accordance with the institutional guidelines.

### Data Acquisition

Magnetic resonance images were obtained on a 3.0T MR scanner (Signa Excite; GE HealthCare, Milwaukee, WI, USA) at Ren Ji Hospital within 2 days after the trauma. A standard head coil was used with foam padding to restrict head motion. DTI data was acquired using a spin-echo single shot echo-planar pulse sequence (TR = 15000ms, TE = 68ms, matrix = 110 × 110, FOV = 220 × 220mm^2^, NEX = 1, slice thickness = 2 mm, gap = 0). The diffusion-sensitizing gradients were applied along 20 non-collinear directions with a b value of 1000 s/mm^2^, together with an acquisition without diffusion weighting (b = 0).

### Data Processing

DTI data were analyzed using DTI-studio (Version 2.40, Department of Radiology, Johns Hopkins University, Baltimore, MD; available at: http://cmrm.med.jhmi.edu/). Before tensor calculation, the echo planar distortion induced by eddy currents was corrected using an algorithm that determines the optimum affine transformation to be applied to each diffusion-weighted image. FA and MD maps were then computed from the DTI data on a pixel by pixel basis according to a well-established method [20]. VBA was performed using the Statistical Parametric Mapping (SPM5, Wellcome Department of Imaging Neuroscience, London, UK; available at http://www.fil.ion.ucl.ac.uk/spm/software/spm5) implemented on MATLAB R2010a (MathWorks Inc., Sherborn, MA, USA). It was implemented as follows: First, a participant-specific b0 template was built with the data from the healthy controls (22 participants), the trauma-exposed victims with PTSD (21 participants) and trauma-exposed victims without PTSD (17 participants). Each b0 volume was normalized to the EPI template provided by SPM5 using a nonlinear coregistration method, with a reslicing resolution of 2 × 2 × 2 mm^3^. Then, the normalized b0 template were averaged and smoothed with an 8 mm full width at half maximum (FWHM) Gaussian kernel to yield the b0 template. Secondly, all original b0 images of each subject were normalized to this b0 template, and the resulting transformation matrix was applied to the FA and MD maps. Finally, all images were smoothed with an 8 mm FWHM isotropic Gaussian kernel to decrease spatial noise and compensate for the inexact nature of normalization. In addition, we generated a WM mask from the b0 template’s image which was segmented using the SPM5 segmentation routine.

### Statistical Analysis

#### Trauma-exposed Victims with PTSD versus Trauma-exposed Victims without PTSD

We performed a whole-brain VBA using the normalized and smoothed FA/MD maps for a 2-sample t-test with SPM5 to identify WM changes from the 21 trauma-exposed victims with PTSD and 17 trauma-exposed victims without PTSD. In addition, because the study was focused on WM changes of trauma-exposed victims, the WM mask generated from the participant-specific b0 template was used to restrict the search volume for statistics. The correction for multiple comparisons was performed using a Monte Carlo simulation. A corrected threshold of *P* < 0.05 was derived from a combined threshold of *P* < 0.001 for each voxel and a cluster size >76 voxels, which was determined by the AlphaSim program in the REST software (parameters: single voxel *P* = 0.001, 5000 simulations, FWHM = 8 mm, cluster connection radius = 5 mm, with WM mask, http://www.restfmri.net/forum/RESTV1.8). Furthermore, the correlation analysis between PTSD symptom severity and FA/MD values was performed using multiple regression in the basic model of the REST. Altered regions of WM tract in group comparison (trauma-exposed victims with PTSD Versus trauma-exposed victims without PTSD) were extracted as ROIs from the FA/MD maps of trauma-exposed victims with PTSD. Symptom severity was represented by the total score received on the CAPS (the composite of frequency and intensity score). Significance level was set to *P* < 0.05 corrected for AlphaSim. 

#### Trauma-exposed Victims with PTSD versus Healthy Controls; Trauma-exposed Victims without PTSD versus Healthy Controls

In order to explore altered WM regions correlating with the effect of the trauma events, contrasts were performed between the 21 trauma-exposed victims with PTSD and 22 healthy controls using a 2-sample t-test with SPM5. A comparison between 17 trauma-exposed victims without PTSD and 22 healthy controls were also conducted. The WM mask was also used to confine the statistic to the white matter region. Significant differences were defined as those which survived a statistical threshold of *P* < 0.05 corrected for AlphaSim.

#### Trauma-exposed Victims with PTSD at Baseline versus Follow-up

A paired t-test was used to identify WM changes in the 11 follow-up scans of trauma-exposed victims with PTSD compared to their baseline DTI scan. The WM mask was used and the thresholds were set at *P* < 0.05 corrected for AlphaSim. The mean FA/MD values of altered regions identified from the paired comparisons were exported from the FA/MD maps of the follow-up scans of trauma-exposed victims with PTSD. A Pearson correlation analysis was performed using SPSS17.0 to explore the correlation between the mean FA/MD values of the ROI and CAPS scores at diagnosis. 

Furthermore, the altered WM regions from the group comparison between trauma-exposed victims with and without PTSD were used as ROIs, and the FA/MD values of these ROIs were exported from their baseline data. Pearson correlations were performed between these FA/MD values exported from the baseline data and the FA/MD values of the regions with significance differences from the above paired comparisons exported from the follow-up data of trauma-exposed victims with PTSD in order to compare the effect of WM changes within 2 days after trauma to the effect of WM changes at diagnosis. 

The follow-up scan of the trauma-exposed victims without PTSD was performed in only 5 subjects who did not develop PTSD. They were not analyzed in this study because data may be misleading due to the small group size.

## Results

### Demographics and Clinical Scores

Pearson chi-square tests for categorical data and Student's t-tests for continuous variables were carried out in SPSS17.0 to evaluate differences in socio-demographic characteristics. The demographic and clinical characteristics of each group are summarized in Table 1. There was no significant difference in age, gender composition or years in education between the trauma-exposed victims with and without PTSD, between the trauma-exposed victims with PTSD and healthy controls, and between the trauma-exposed victims without PTSD and healthy controls. As expected, the trauma-exposed victims with PTSD had higher scores on the CAPS and ASDI than the trauma-exposed victims without PTSD.

**Table 1:** Demographic and clinical information for trauma-exposed victims with and without PTSD, follow-up of trauma-exposed victims with PTSD, and healthy controls.

	Trauma-exposed victims with PTSD (N=21)	Trauma-exposed victims without PTSD (N=17)	P	Follow-up of trauma-exposed victims with PTSD (N=11)	Healthy controls (N=22)	P*	P**	P***
Age(years)	40.86±12.26	35.64±11.91	0.53	42.09±12.79	40.23±12.54	0.53	0.76	0.43
Gender (M/F)	8/13	11/6	0.10	4/7	9/13	0.10	0.72	0.47
Educations (years)	11.24±3.60	12.71±4.06	0.40	10.09±3.91	14.05±2.59	0.40	0.65	0.95
ASDI (2 days post-accident)	15.48±5.51	8.76±3.13	<0.001			<0.001		
CAPS (1 months post-accident)	39.95±19.48	10.89±5.27	<0.001			<0.001		
CAPS (6 months post-accident)	33.48±14.32	8.26±3.98	<0.001			<0.001		
CAPS (at diagnosis)	44.00±14.92			40.82±16.64				
Mean time from accident to diagnosis (months)	2.24±2.00			4.18±2.49				

* for Trauma-exposed victims with PTSD versus Trauma-exposed victims without PTSD.

** for Trauma-exposed victims with PTSD versus Healthy controls.

*** for Trauma-exposed victims without PTSD versus Healthy controls.

PTSD = posttraumatic stress disorder; ASDI = acute stress disorder inventory; CAPS = clinician-administered PTSD Scale.

### Trauma-exposed Victims with PTSD versus Trauma-exposed Victims without PTSD

**Figure 1:**
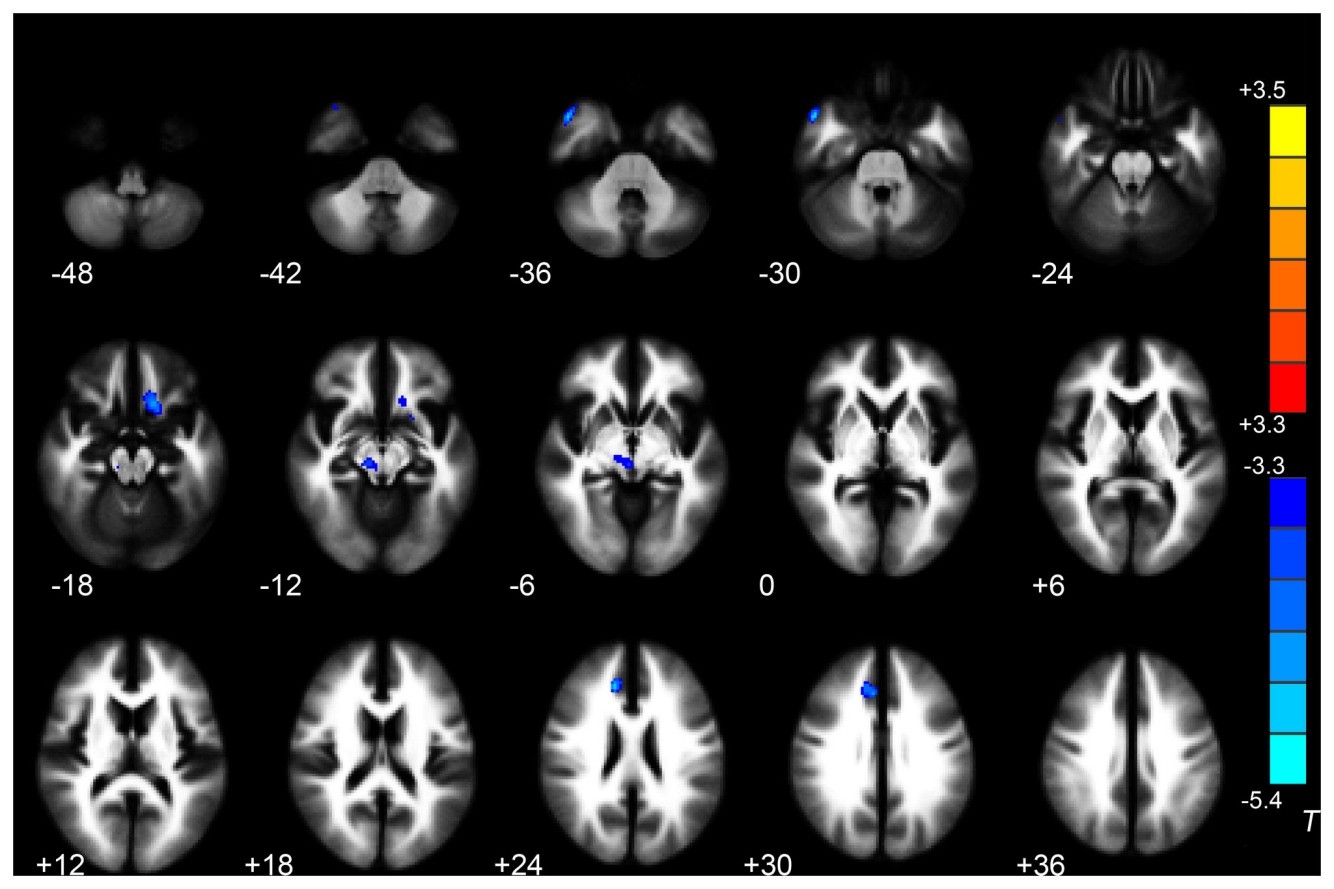
Significant differences in FA values between trauma-exposed victims with and without PTSD. These clusters were shown in the WM of right anterior cingulate cortex, right middle temporal gyrus, right midbrain, and left gyrus rectus/medial orbitofrontal gyrus (2-sample t-test, *P* < 0.05, AlphaSim-corrected). Cold colors indicate decreases in FA. The left part of the figure represents the participant’s right side. PTSD = posttraumatic stress disorder; FA = fractional anisotropy; WM = white matter.

**Figure 2:**
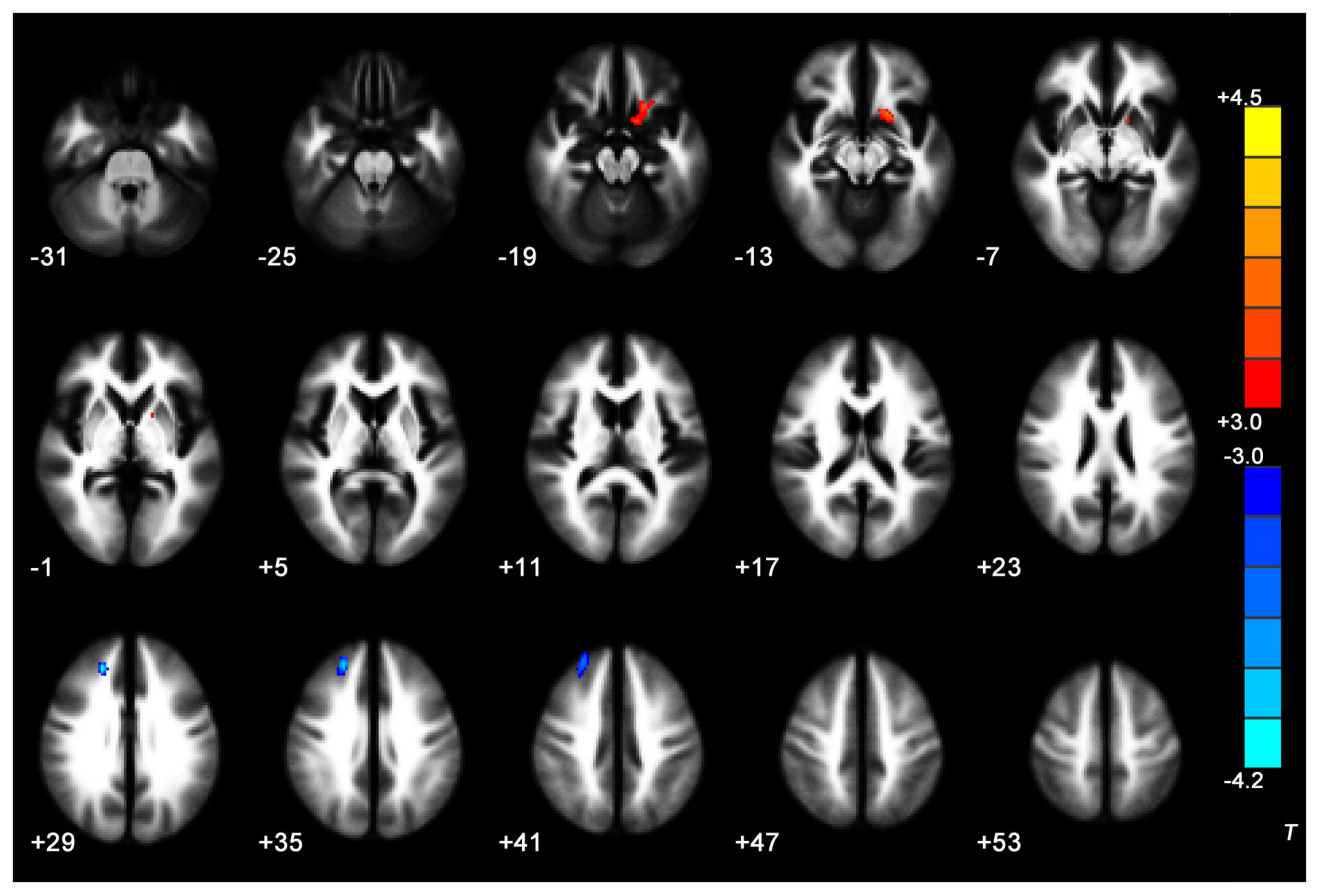
Significant differences in MD values between trauma-exposed victims with and without PTSD. These clusters were shown in the WM of right superior frontal gyrus, and subcallosal gyrus (2-sample t-test, *P* < 0.05, AlphaSim-corrected). Cold colors indicate decreases in MD and warm colors indicate increases in MD. The left part of the figure represents the participant’s right side. PTSD = posttraumatic stress disorder; MD = mean diffusivity; WM = white matter.

**Figure 3:**
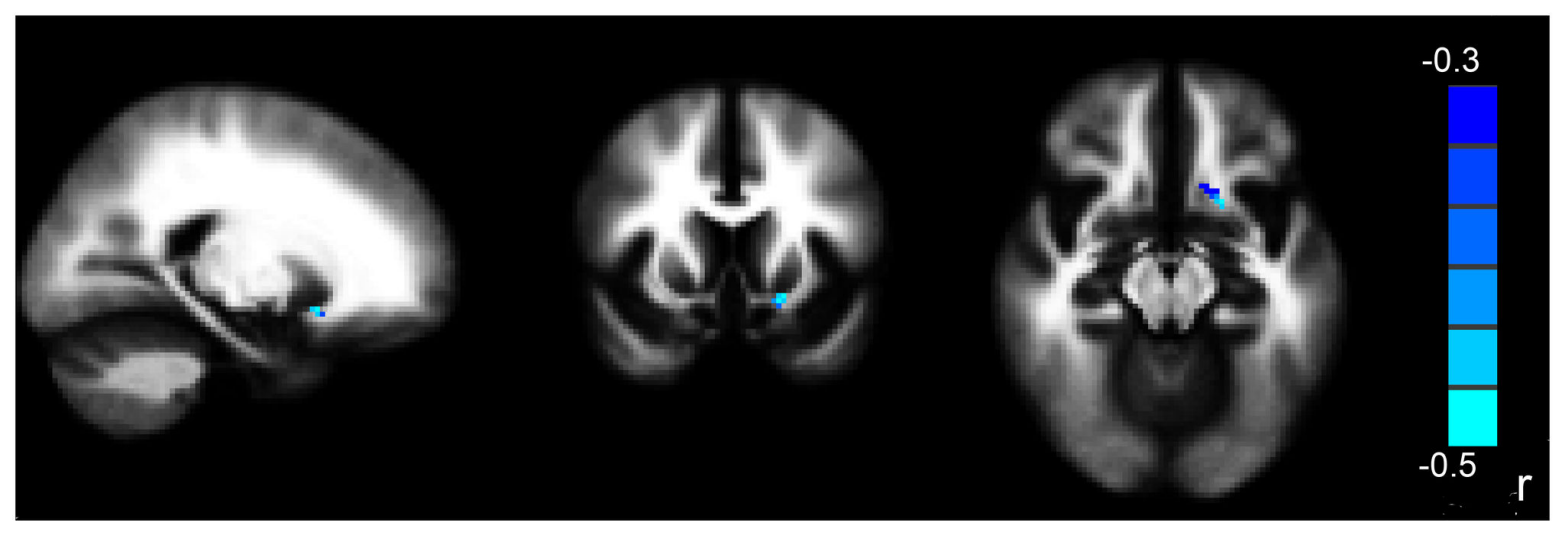
The mean FA values in the left vmPFC in trauma-exposed victims diagnosed with PTSD within 1 or 6 months was negatively correlated with their CAPS scores at diagnosis (***P* < 0.05 corrected for AlphaSim)**. The right part of the figure represents the patient’s left side. CAPS = clinician administered PTSD scale; PTSD = post-traumatic stress disorder; FA = fractional anisotropy; vmPFC = ventromedial prefrontal cortex.

VBA results showed that the trauma-exposed victims with PTSD had decreased FA values in WM of the right ACC, right middle temporal gyrus, right midbrain and left gyrus rectus/medial OFC. No WM regions in the trauma-exposed victims with PTSD had significantly higher FA values than in the trauma-exposed victims without PTSD (*P* < 0.05 corrected for AlphaSim) (Table 2, Figure 1). The trauma-exposed victims with PTSD also had decreased MD in WM of the right superior frontal gyrus, and increased MD in WM of the left subcallosal gyrus (P < 0.05 corrected for AlphaSim) (Table 2, Figure 2). The mean FA values derived from ROI analysis of the left gyrus rectus/medial OFC (the peak MNI coordinate: -20, 12, -14) tended to correlate negatively with the CAPS score in the trauma-exposed victims with PTSD at the time of diagnosis (Figure 3). There was no correlation between the MD values in WM of right superior frontal gyrus and left subcallosal gyrus from the MD maps of trauma-exposed victims with PTSD and CAPS scores. 

**Table 2:** Significant between-group differences in FA/MD values in trauma-exposed victims with and without PTSD (*P* < 0.05, AlphaSim corrected).

Diffusion parameter	Region of white matter	Peak MNI coordinate			Number of cluster voxels	Peak T value
		x	y	z		
FA	R-middle temporal gyrus WM	50	10	-32	126	-5.46
	R-anterior cingulate cortex WM	14	30	26	117	-5.38
	R-midbrain WM	12	-24	-10	121	-3.86
	L- gyrus rectus/medial OFC WM	-16	18	-16	158	-4.75
MD	R-superior frontal gyrus WM	22	38	32	189	-4.52
	L-subcallosal gyrus WM	-18	12	-12	171	3.65

R = right; L = left; PTSD = posttraumatic stress disorder; FA = fractional anisotropy; MD = mean diffusivity.

T < 0 indicates decreased FA/MD values in trauma-exposed victims with PTSD.

T > 0 indicates increased MD values in trauma-exposed victims with PTSD.

### Trauma-exposed Victims with PTSD versus Healthy Controls

**Figure 4:**
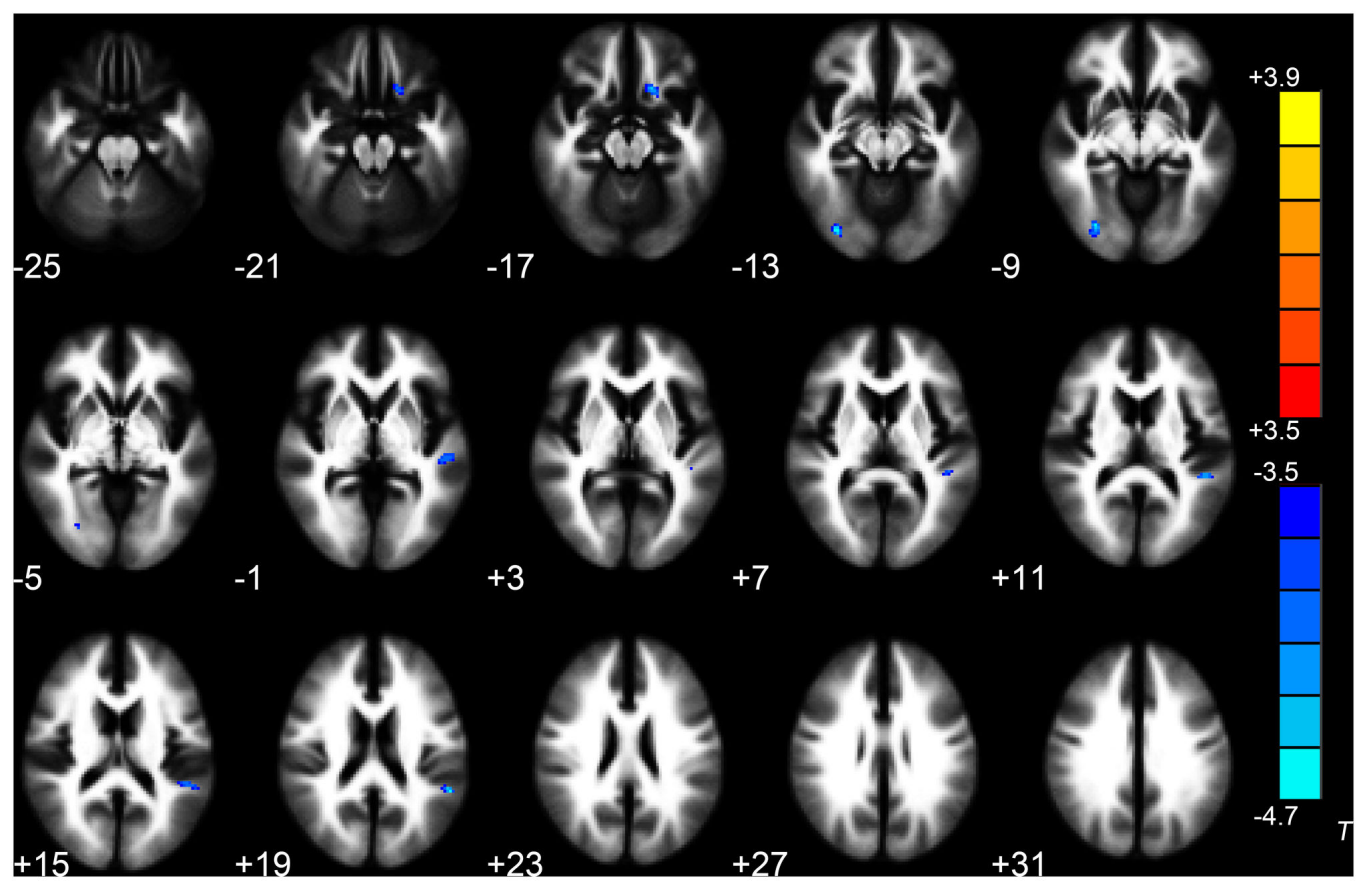
Significant differences in FA values between trauma-exposed victims with PTSD and healthy controls These clusters were shown in the WM of right inferior occipital gyrus, left superior temporal gyrus and left superior frontal gyrus, orbital part (**2-sample t-test, *P* < 0.05, AlphaSim-corrected)**. Cold colors indicate decreases in FA. The left part of the figure represents the participant’s right side. PTSD = posttraumatic stress disorder; FA = fractional anisotropy; WM = white matter.

Across the whole brain volume, we found an FA decrease at *P* < 0.05 (AlphaSim corrected) in the WM of right inferior occipital gyrus, left superior temporal gyrus and left superior frontal gyrus, orbital part (Table 3, Figure 4). No significant differences was observed in MD between trauma-exposed victims with PTSD in baseline scans and the healthy controls (*P* < 0.05 corrected for AlphaSim). 

**Table 3:** Significant between-group differences in FA values in trauma-exposed victims with PTSD and healthy controls (*P* < 0.05, AlphaSim-corrected).

Diffusion parameter	Region of white matter	Peak MNI coordinate			Number of cluster voxels	Peak T value
		x	y	z		
FA	L-superior frontal gyrus, orbital part WM	-16	20	-18	79	-4.689
	L- superior temporal gyrus WM	-54	-44	18	142	-4.935
	R- inferior occipital gyrus WM	34	-82	-12	87	-4.944

L = left; FA = fractional anisotropy.

T < 0 indicates decreased FA values in trauma-exposed victims with PTSD

### Trauma-exposed Victims without PTSD versus Healthy Controls

No significant FA or MD differences were found in the comparison between trauma-exposed victims without PTSD and healthy controls (*P* < 0.05 corrected for AlphaSim).

### Trauma-exposed Victims with PTSD at Baseline versus Follow-up

**Figure 5:**
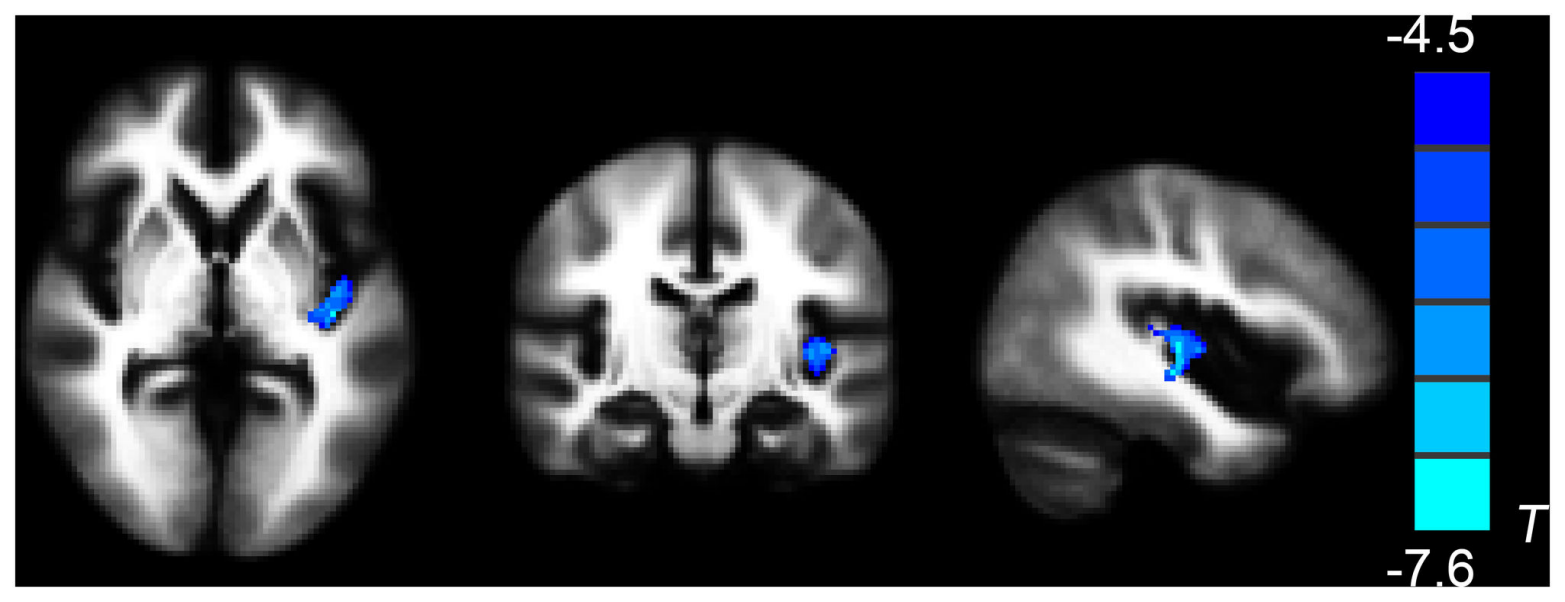
The follow-up scans of trauma-exposed victims with PTSD showed decreased FA in left insula WM compared with their baseline DTI scans (**paired t-sample t-test, *P* < 0.05, AlphaSim-corrected)**. Cold colors indicate decreases in FA. The left part of the figure represents the participant’s right side. PTSD = posttraumatic stress disorder; FA = fractional anisotropy.

Decreased FA was found in the left insula in the follow-up scans of trauma-exposed victims with PTSD in relation to their baseline DTI scan (*P* < 0.05 corrected for AlphaSim) (Table 4, Figure 5), but no significant differences were detected for MD. In addition, there was no correlation between the CAPS scores and FA values of the insula in follow-up scans of trauma-exposed victims with PTSD (r = 0.488, *P* = 0.128).

**Table 4:** Areas of decreased FA in follow-up scans of trauma-exposed victims with PTSD compared with the baseline scan (*P* < 0.05, AlphaSim-corrected).

Diffusion parameter	Region of white matter	Peak MNI coordinate			Number of cluster voxels	Peak T value
		x	y	z		
FA	L- insula WM	-38	-22	-6	217	-7.601

L = left; FA = fractional anisotropy.

T < 0 indicates decreased FA values in follow-up scans of trauma-exposed victims with PTSD.

**Figure 6:**
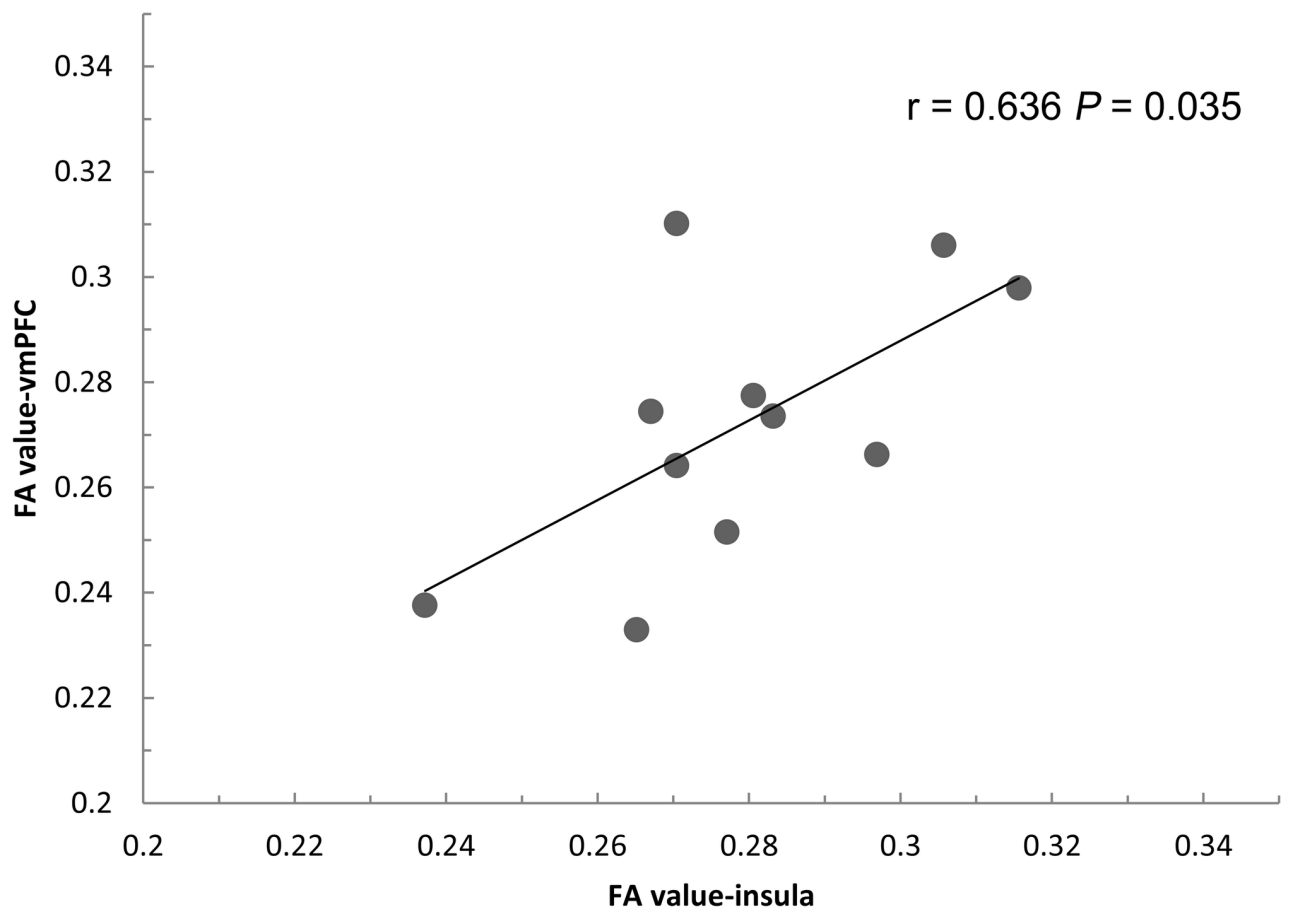
Scatter plot illustrating the relationship between the mean FA values in the insula in the follow-up scan of trauma-exposed victims with PTSD and the mean FA values in the vmPFC in their baseline DTI scans. FA = fractional anisotropy; vmPFC = ventromedial prefrontal cortex.

The decreased FA values in the left insula (the altered region from paired comparison) exported from 11 follow-up scans of trauma-exposed victims with PTSD were found to correlate positively with the decreased FA values in left gyrus rectus/medial OFC (the altered region from the group comparison between trauma-exposed victims with and without PTSD) from their baseline DTI scan (r = 0.636, *P* = 0.035, Figure 6). There was no correlation with FA values in the right anterior cingulate cortex (r = -0.052, *P* = 0.880), right middle temporal gyrus (r = 0.343, *P* = 0.301), or right midbrain (r = -0.237, *P* = 0.482). 

## Discussion

The main aims of this study were to investigate the possible WM microstructure alterations before diagnosis as susceptibility factors in trauma-exposed victims who go on to develop PTSD and to examine the ability of these susceptibility factors to predict the course of longitudinal PTSD. The present study demonstrated that, compared with trauma-exposed victims without PTSD, trauma-exposed victims with PTSD showed decreased FA in WM of the ACC, left gyrus rectus/medial OFC, right middle temporal gyrus and midbrain, and increased MD in WM of the left subcallosal gyrus within 2 days following a traffic accident. Our hypothesis was that alterations in brain WM microstructure, particularly in vmPFC regions, might be detectable through the use of neuroimaging on people at risk of developing PTSD. In addition, we also found decreased FA values in the left insula, a possible acquired sign of PTSD, in the follow-up scans of trauma-exposed victims with PTSD, which correlated positively with their decreased FA values of vmPFC region in the baseline scan.

The current results in WM of the ACC, middle temporal gyrus, vmPFC and midbrain are in part consistent with several previous DTI studies in PTSD. The previous studies found WM hypointensity at the level of the cingulum bundle [6,23], corpus callosum [24,25], temporal lobe [26], prefrontal cortical [6,27] and midbrain [18] in adults or children with PTSD. A tract-based spatial statistics (TBSS) study found a systematic pattern of FA reduction, implying compromised integrity of neural pathways that involve prefrontal and anterior cingulate regions [27]. In VBA studies, PTSD patients exhibited abnormal WM integrity of the ACC [28,29], and PTSD symptom severity was found to negatively correlate with the extent to which FA values decreased in the ACC [30]. Within 25 days after the traumatic event, significantly decreased FA values in the prefrontal-limbic system were found in earthquake survivors [27]. Moreover, a prospective longitudinal study suggested that combat stress reduced midbrain WM integrity [18].

Our results demonstrated that altered WM microstructures can be identified within 2 days after trauma exposure in patients who go on to develop PTSD. Smaller GMV in the ACC was previously identified as a pre-trauma vulnerability factor for developing PTSD [13]; all participants in that study had undergone the first MRI within 2 years before the earthquake. However, the current results in WM of right ACC, right middle temporal gyrus, and left vmPFC were found 2 days after trauma. It was hard to collect the MR data before these victims were exposed to the trauma, so it was difficult to determine whether or not the results were trauma event-induced. To address this issue, healthy controls were recruited in our study because both genetic and environmental factors have been established as risk factors in PTSD [31]. People in the healthy control group have the same chance of acquiring risk factors, as well as the other factors which might also induce alteration in the WM microstructures, e.g. skill [32], and age [33]. In the current study, no significant differences in FA measures were observed between trauma-exposed victims without PTSD in baseline scans and healthy controls. As the healthy controls were not affected by trauma, it is posited that the WM alterations observed within 2 days of trauma between trauma-exposed victims with and without PTSD may not correlate with the effect of the trauma events. Specifically, due to the fact that we found an FA decrease in the WM of vmPFC region in the comparison between trauma-exposed victims with PTSD in baseline scans and healthy controls as well, the alterations in WM microstructure of vmPFC region could be a susceptibility factors. Moreover, a previous study has suggested that the low expressing 5-HTTLPR variant is a significant genetic risk factor for PTSD development after severe trauma exposure [34]. The 5-HTTLPR constitutes a genetic candidate region that may modulate emotional responses to traumatic events. The interaction between variations of 5-HTTLPR and stressful life events could well be predictors of PTSD. The 5-HTTLPR genotype alters resting brain function in emotion-related regions, including the amygdala and vmPFC [35]. A recent study showed that the 5-HTTLPR genotype appeared to influence the WM microstructure of the left frontal uncinate fasciculus [36], which overlapped with the vmPFC region in our study. We suggest that alterations in brain WM microstructure that are found within 2 days of experiencing a traumatic event, particularly in the vmPFC region, may be associated with genetic susceptibility, and may be a possible pre-existing vulnerability factor for the development of PTSD following trauma exposure. 

The current study also found a significant negative correlation between altered WM microstructure in the left vmPFC and the severity of PTSD. The vmPFC abnormalities in PTSD are of particular interest and often mentioned in the pathophysiology of PTSD, because this region is thought to mediate the extinction of conditioned fear and the volitional regulation of negative emotion. It has been theorized that the vmPFC exerts inhibition on the amygdala and that a defect in this inhibition could account for the symptoms of PTSD [37]. A meta-analysis of functional studies of PTSD demonstrated hypoactivity in the vmPFC but hyperactivity in the amygdala [5]. Hypoactivation of the vmPFC in PTSD patients reflected a deficit in reflexive emotion regulation processes occurring in the absence of self-reflection about emotion or deliberate attempts at emotional control and was reflected clinically in emotional dysregulation symptoms and generalized anxiety. Our findings show decreased FA values and increased MD in the left vmPFC region. The main determinant of FA value is the architecture of the myelin, so decreased FA is likely to reflect the disruption of integrity of the nerve-sheath in WM [38]. MD indexes the overall presence of WM. FA and MD may be complementary indices for the density and integrity of WM [38]. Dense WM connections were found between the vmPFC and amygdala, facilitating bidirectional communication between these areas [39]. As the vmPFC is involved in processing of fear and anxiety, our results suggest that the abnormal WM microstructures could predict future PTSD symptoms. At the resting-state functional level, we have previously shown altered FC readings in the amygdala in PTSD patients [16]. We now hypothesize that these changes may be caused by defective inhibition from abnormal WM in the vmPFC region. Considering that altered FC of the PCC with the hippocampus was found in the functional study, rather than in the DTI study [16], we hypothesized that WM changes in the hippocampus could be stress-induced. To support our hypothesis, Admon and colleagues demonstrated that a reduction in hippocampal volume and connectivity with the vmPFC following exposure to repeated military stress were related to increased PTSD-related symptoms, and may indicate a maladaptive response to such real life stressors [40].

We found abnormal WM microstructures of both midbrain and vmPFC within 2 days of the traumatic event. A recent study demonstrated that combat stress reduced midbrain activity and integrity [18]. However, no significant differences were observed in the midbrain or PFC in the combat group before stress. The PFC-midbrain pathway plays a central role in regulating the firing pattern of dopamine (DA) neurons. Chronic stress induces working memory impairment through reducing the dopamine turnover in terminal regions in the PFC [41]. Catecholamine-O-methyltransferase (COMT) regulating prefrontal dopamine turnover predicted reduced DA synthesis in midbrain and qualitatively affected the interaction with PFC, suggesting a systems-level mechanism for cognitive and neuropsychiatric impairment [42]. In our current study, decreased WM integrity was found in the vmPFC within 2 days of a traumatic event, which may have a correlation with the abnormal WM of midbrain. A further study is needed to determine why these WM changes were found within 2 days of a traumatic event.

Left insula WM changes were found to be an acquired sign of PTSD in the follow-up group. In previous studies, smaller insula volumes were found to correlate with PTSD symptoms induced by military combat [43]. The gray matter density of the insula negatively correlated with re-experiencing trauma and flashbacks [44,45]. Another study provided evidence of structural and functional abnormalities of the insula in patients with PTSD 6 months after experiencing a fire disaster [46]. The fMRI findings from this study showed that decreased insula activation may be involved in the declarative memory deficits associated with PTSD, which might be implicated in the symptom generation of PTSD [46]. Furthermore, the GMV of bilateral insula was greatly decreased in patients with PTSD [46]. Our present study also found decreased FA values in the insula, which positively correlated with the FA value decreases in the vmPFC of their baseline DTI scan, in trauma-exposed victims who developed PTSD. The OFC and the prefrontal cortex, and the limbic system and the temporal lobe have connections with the insular lobe [47]. The insula, OFC and temporal pole constitute the network which plays a prominent role in establishing balance between experience, affect, and behavior [48]. The vmPFC is linked to the experience and regulation of emotion [49] and the insula is linked to negative emotional responses [50], and to emotional recall [49]. However, the exact reason why the lowered FA values in the insula correlated positively with those in the vmPFC of the baseline scan in trauma-exposed victims who developed PTSD is unclear and a further study is needed. In addition, in our functional study, increased connectivity was found in the left insula in the PTSD group within 2 days of the accident [15]. The present study cannot draw definite conclusions as to whether functional defects of insula precede the structural changes, which clearly necessitates future studies using a large sample study.

The current study still has a number of limitations. First, the study design did not allow the differentiation between pre-existent vulnerability factors and acquired signs, as the DTI data from both the trauma-exposed victims with and without PTSD were collected within 2 days after trauma exposure. Although the healthy controls were recruited to exclude the correlation of WM alterations with the effect of the trauma events, it was still difficult to ensure that our results showed susceptibility factors for PTSD. The second limitation is the relatively small sample size of the follow-up group, especially the follow-up of trauma-exposed victims without PTSD. Third, the VBA method was limited by the low spatial resolution, especially when isotropic spatial smoothing was applied. Fourth, in a small number of cases, PTSD symptoms may not appear until years after the traumatic event. Six months may be too short for the diagnosis of the trauma-exposed victims without PTSD. Our research group will continue to follow up the participants for a further study. Fifth, the difference between our findings and the previous report is that the CAPS score in the current study (44.00±14.92) was much lower than the score produced in previous PTSD studies. The possible explanation could be that the traffic accident-induced stresses reported in our study were not as serious as those which featured in the previous studies. The victims in our study experienced, witnessed, or were confronted mostly with car accidents. 

## Conclusions

Despite these limitations, altered WM microstructure was identified within 2 days after trauma in the individuals who developed PTSD at a later date. A reduction in FA values in the insular region in the follow up scan of victims with PTSD could be an acquired sign of PTSD. The decreased FA values and increased MD values found at baseline in the vmPFC region could predict the future severity of PTSD and the acquired signs, suggesting that these imaging results may be vulnerability markers showing predisposition towards developing PTSD. Our findings provide a better understanding of the neurological underpinnings of the early stage of the disease and may contribute towards the development of effective methods of preventing PTSD.
